# A case study of risk factors for lymphatic filariasis in the Republic of Congo

**DOI:** 10.1186/1756-3305-7-300

**Published:** 2014-07-01

**Authors:** Cédric B Chesnais, François Missamou, Sébastien D Pion, Jean Bopda, Frédéric Louya, Andrew C Majewski, Peter U Fischer, Gary J Weil, Michel Boussinesq

**Affiliations:** 1 UMI 233, Institut de Recherche pour le Développement (IRD) and University of Montpellier 1, 911 avenue Agropolis, P.O. Box 64501, 34394 Montpellier, Cedex 5, France; 2 Programme National de Lutte contre l’Onchocercose, Direction de l’Epidémiologie et de la Lutte contre la Maladie, Ministère de la santé et de la Population, BP 1066 Brazzaville, Republic of Congo; 3 Centre for Research on Filariasis and other Tropical Diseases (CRFilMT), P.O. Box 5797, Yaoundé, Cameroon; 4 Infectious Diseases Division, Department of Internal Medicine, Campus Box 8051, Washington University School of Medicine, 660 S. Euclid Ave., St. Louis, MO, USA

**Keywords:** Africa, Congo, Filariasis, Epidemiology, Risk factors, Bed nets, Community study, Lymphatic filariasis

## Abstract

**Background:**

Little is known regarding risk factors for lymphatic filariasis (LF) in Central Africa. We studied the epidemiology of LF in an endemic village in the Republic of Congo.

**Methods:**

Dependent variables were *Wuchereria bancrofti* antigenemia (ICT card test) and microfilaremia (night blood smears). The following factors were investigated: sex, age, bed net, latrines, source of water, uptake of anthelmintic drugs, hunting/fishing activities, and occasionally sleeping in the bush. Mixed multivariate logistic regression models were used.

**Results:**

134 of 774 subjects aged ≥ 5 years (17.3%) had *W. bancrofti* antigenemia and 41 (5.3%) had microfilaremia (mf). Infection rates increased with age up to roughly 20 years and remained stable thereafter. Multivariate analysis of antigenemia demonstrated an increased risk for males (OR = 2.0 [1.3-3.0]) and for people who hunt or fish (OR = 1.5 [1.0-2.4]) and a protective effect of latrines (OR = 0.5 [0.4-0.8]). Among males, those hunting or fishing at night had an increased risk for antigenemia (OR = 1.9 [1.1-3.5]), and use of latrines was protective (OR = 0.5 [0.3-0.9]). For females, bed nets were protective (OR = 0.4 [0.1-0.9]), and there was a strong household effect (intraclass correlation coefficient [ICC]: 0.24). When mf was used as the dependent variable, males had a higher risk for infection (OR = 5.4 [2.1-13.4]), latrines had a protective effect (OR = 0.4 [0.1-0.9]) and there was a marked household effect (ICC = 0.49).

**Conclusions:**

Age, sex, and occupation-dependent exposure to mosquitoes were important risk factors for infection with *W. bancrofti* in this study. It is likely that men often acquire infection in high transmission areas outside of the village, while children and women are infected in areas with lower transmission inside or near the village. Additional studies are needed to determine whether these findings apply to other areas in Central Africa.

## Background

Fourteen years after its launch in 2000, the Global Programme to Eliminate Lymphatic Filariasis (GPELF) is well under way. For example, in 2012 (the last year with reported data), 56 of 73 LF-endemic countries provided mass drug administration (MDA) to approximately 425 million people [1]. Despite a number of challenges, GPELF is progressing in many areas in West and East Africa, and some African countries are already approaching elimination targets [2]. In contrast, lymphatic filariasis (LF) elimination programs are not as advanced in Central Africa, and there are three main reasons for this. First, relatively few epidemiological surveys for LF were conducted in Central African countries before 2005 [3-6], and the geographical distribution of LF is still not well defined in this region. Second, the presence of loiasis in many areas of Central Africa [7] has delayed implementation of MDA with ivermectin (IVM) and albendazole (ALB) because of the risk of post-IVM serious adverse events [8]. Third, many countries in Central Africa face special challenges in implementing large scale NTD control and elimination programs because of limited financial resources, ongoing or recent armed conflicts, and insecurity.

Since 2005, the World Health Organization (WHO) and several research teams have conducted prevalence surveys for LF in Central African countries using an immunochromatographic card test (ICT) to detect circulating filarial antigens (CFA), and/or night blood smears for detection of *Wuchereria bancrofti* microfilariae (mf) [9]. High infection rates reported from these surveys have not been confirmed by subsequent investigations in some cases (authors’ unpublished observations). Mapping studies and historical observations suggest that while LF is present in Central Africa, endemicity rates are usually low, and the distribution of the infection is highly focal [10]. Little is known regarding risk factors or transmission parameters that contribute to this focality, and such information might be useful for planning control programs. This paper reports results of a case study of the epidemiology of LF in an endemic village in the Republic of Congo that was identified during surveys of bancroftian filariasis in that country.

## Methods

### Context of the study and selection of study community

The study was carried out as part of the project called “Death to Onchocerciasis and Lymphatic Filariasis” (DOLF), which aims to improve MDA programs for LF and onchocerciasis (http://www.dolf.wustl.edu). One of the arms of this project consists in performing community trials to assess the impact of six-monthly treatments with ALB alone on LF and soil-transmitted helminths. Albendazole MDA could be a safe alternative to IVM + ALB for LF elimination in areas with coendemic loiasis. Community surveys (convenience samples of approximately 100 adults per village) were performed in 40 villages in Niari and Bouenza divisions in the Republic of Congo in 2010-2011 to identify suitable sites for a community MDA study. Filarial antigenemia rates ranged from 0 to 23.9% in these villages. The village with the highest rate was Séké Pembé [11]; this village was selected for the community trial, and for the present epidemiologic study.

### Study design

#### Study site

**Figure 1:**
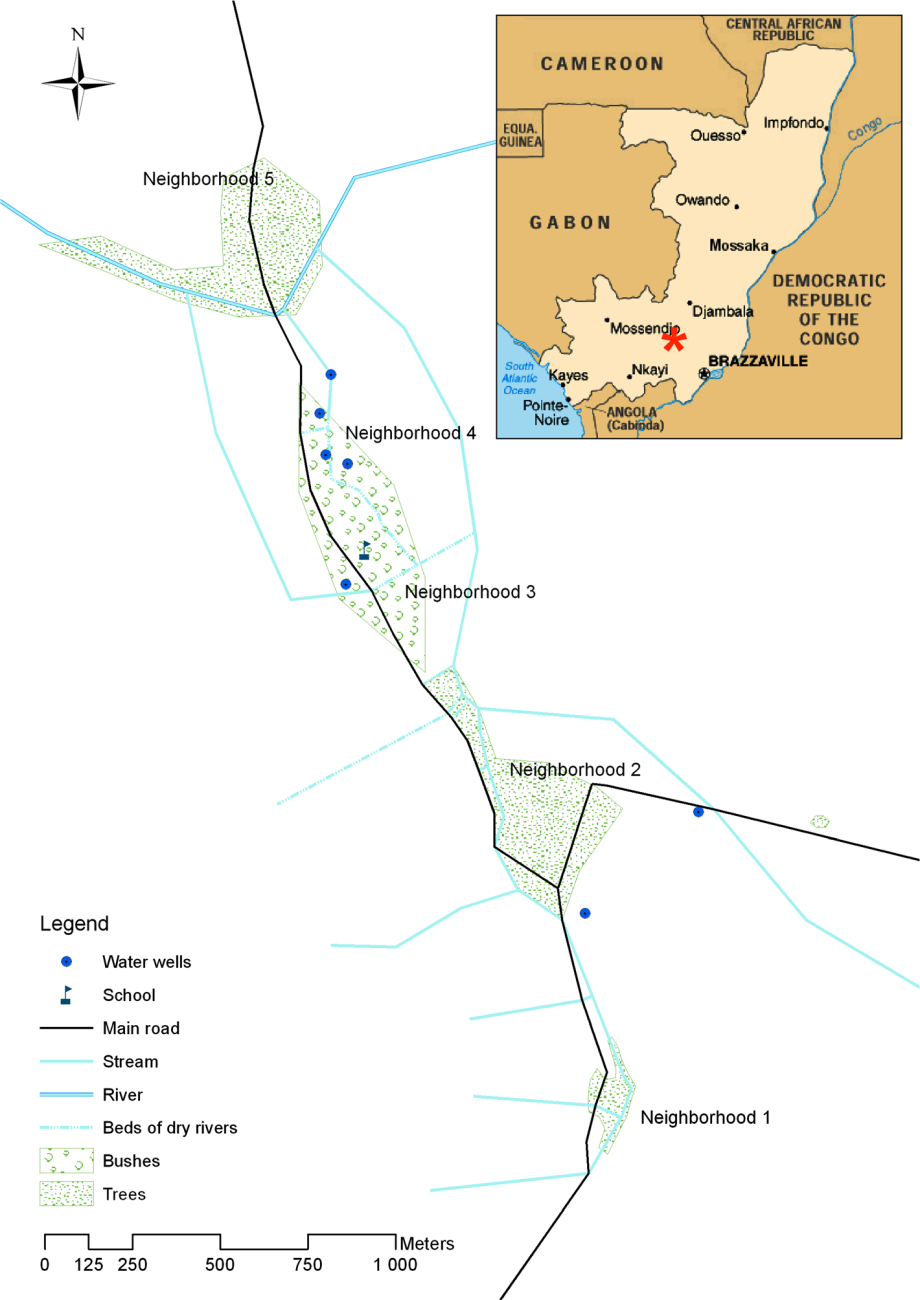
**Study area.** Red star indicates location of Séké Pembé.

Séké Pembé village (4°04′S, 13°31′E; elevation 200 meters) is located in a well-drained, non-arid, savannah area. Seasonality is well marked with a long dry season (May to September), a long rainy season (October to December), a short dry season (January to February), and a short rainy season (March to April). The rainfall is 1000 to 1300 mm per year. The village is 5 km away from the forest fringe and 20 km away from densely forested areas. Séké Pembé is relatively large (10 km^2^) compared to other villages in the region. The population density in the village (105.5 inhabitants per km^2^) is high in a country with a very low population density (13 inhabitants per km^2^). Houses in the village are distributed in five neighborhoods along a road that connects two district capitals. There is a small permanent river flowing between two of the neighborhoods, a stream that meanders along the main road, and smaller streams that are dry during the dry season (Figure 1). All adults and older children perform farm work. Some residents also hunt and/or fish regularly. All houses have terracotta walls and a corrugated metal roof. Houses do not have running water or indoor toilets.

### Procedures

A complete census of the village was performed in August 2012, and blood samples were collected in September-October 2012. Consenting adults and assenting children aged ≥ 5 years were enrolled in the study. Screening for LF was performed with a rapid test for filarial antigenemia (see below). Subjects with negative antigen tests were treated with ALB immediately after testing. Individuals with positive antigen tests were asked to return to the testing station for microfilaria testing between 10:00 PM and midnight*.* These individuals were treated with ALB just after the second blood sampling. Pregnant women were not treated; they were asked to visit the village nurse after delivery in order to receive the ALB treatment.

### Questionnaire

A standardized questionnaire administered to each subject collected demographic information (full name, age, sex, name of the head of household), information on the domestic and peridomestic environment, and information on personal activities outside of the peridomestic area that might be related to exposure to mosquito bites. Parents answered the questionnaire for children below 10 years and for older children in some cases. Data were collected using cell phones and the LINKS system [12]. The questionnaire was administered in French or in the local language (Kituba) for those who did not understand French. Questions related to the environment concerned access to a private outside latrine (yes/no), sources of water (river or well), and use of bed nets during the previous night (yes/no), the latter variable being considered as a proxy for regular bed net usage. Questions about activities included the distance (in kilometers) between the subject’s residential compound and their farming site, whether they hunted or fished regularly (yes/no), and whether they sometimes sleep outdoors in the bush (yes/no). In addition, all individuals were asked whether they had taken anthelmintic drugs within the previous year (yes/no).

### ICT test

Capillary blood was obtained by finger prick using a sterile disposable lancet, and the blood was collected in a hematocrit tube. The blood (100 μL) was then transferred to sample application pad on a Binax Filariasis Now card test (Alere, Scarborough, Maine) following the manufacturer’s instructions. A single trained physician (author MB) read all of the tests at 10 minutes, and results were recorded as positive or negative.

### Assessment of microfilaremia in ICT-positive individuals

Two 70 μL thick blood smears were prepared for each person with capillary blood collected by fingerprick between the 22:00 and 0:00 hours. Slides were dehemoglobinized, stained with Giemsa within 24 hours, and read by two experienced microscopists (authors MB and JB). Subjects were considered to have microfilaremia if *W. bancrofti* mf were present on either slide. Slides were read again in cases where mf counts for the two slides from the same subject differed by more than 10%. The mean count from the two slides from each positive subject was used for calculation of mf density (expressed in mf/70 μL).

### Statistical analysis

#### Dependent variables

Two separate analyses were performed with either filarial antigenemia or microfilaremia as dependent variables of interest.

#### Explanatory variables potentially associated with *W. bancrofti* infection

The variables “latrines”, “water source”, “use of bed net”, “anthelmintic treatment within the previous year”, “regular hunting/fishing” and “occasionally sleeping outdoors” were coded as binary variables. Distance between the household and the main agricultural field was considered as a continuous variable. Age was categorized into 4 classes with similar numbers of subjects: 5-10, 11-23, 24-40 and > 40 years. Odds ratio calculations considered 11-23 years as the reference group (OR = 1). The analysis strategy took into account the strong correlation between three variables: sex, “hunting/fishing” and/or “occasionally sleeping in the bush”. Only 8 females out of 420 (1.9%) reported sleeping in the bush vs. 33.5% for males, and none of these females had positive filarial antigen tests. Therefore we stratified the analyses based on sex and developed three different models, one for the total population, and one for each sex. Covariates included in the models for the total and the female populations were “age”, “hunting/fishing”, “latrines”, “bed net”, “water source”, “previous anthelmintic treatment” (with one or more of mebendazole, levamisole, IVM, or ALB), and “distance to field”. “Age”, “hunting/fishing”, “occasionally sleeping in the bush”, “latrines”, “bed net”, “water source”, “anthelmintic intake”, and “distance to the field” were used as covariates for the male population model.

#### Univariate tests, and model description

For categorical variables, χ^2^ tests were performed to assess differences between ICT-positive and ICT-negative subjects and between microfilaremic and amicrofilaremic subjects. The non-parametric Mann-Whitney test and the Cuzick test for trend were used for group comparisons with continuous variables (age, distance to field, and mf density).

All variables with a *P* value for association < 0.25 were included in a full model, and a backward manual stepwise procedure was performed. Mixed multivariate logistic regression models were used to assess associations with ICT and mf results. Household was set as a random effect in both models. Model selection used likelihood-ratio tests for the backward procedure on variables and for the significance of the random effect. Lastly, possible complex interactions were considered including 3-ways interactions between sex, age and the other explanatory variables and 2-ways interactions between latrine and gender, bed net and sex, and bed net and age. All analyses were performed using STATA 12.1 (StataCorp, Texas, USA).

### Ethical clearance

This study was approved by the Ethics Committee for Research in Health Sciences of the Republic of Congo. Congolese members of the research team met with village leaders to explain the purpose of the study, and they later explained the study to all participants. Adult participants signed an informed consent form. Participants younger than 18 years of age were enrolled only if they expressed verbal assent to participate in the study and if at least one parent signed a consent form.

## Results

### Demographic characteristics

In August 2012, a total of 1,055 individuals were recorded in Séké Pembé, including 876 individuals aged ≥ 5 years. Among the latter, 54 were absent from the village during the study, 20 refused to participate, 8 were ill, 10 had permanently left the village since the census, and 10 were pregnant women who also refused to participate in the study. Thus, 774 individuals (88.3% of those aged ≥ 5 years) with 354 males (45.7% of the total studied population) and 420 females from 248 households were included in the study. The median age of the studied population was 23 years (range: 5-92, interquartile range: 10-40). Mean age and sex ratios were similar in participants and non-participants. Parasitological results according to age and sex are presented in Table 1, and results of univariate analysis of potential risk factors for *W. bancrofti* infection*s* are summarized in Tables 2 and 3.

**Table 1:** ***Wuchereria bancrofti *** **prevalence and intensity of infection in Séké Pembé**

	**Total**					**Male**					**Female**				
	**N**	**ICT-positive (%)**	**mf positive (%)**	W. bancrofti mf count* per 70 μL		**N**	**ICT-positive (%)**	**mf positive (%)**	W. bancrofti mf count* per 70 μL		**N**	**ICT-positive (%)**	**mf positive (%)**	W. bancrofti mf count* per 70 μL	
				**GM**^**#**^	**Median (range)**				**GM**^**#**^	**Median (range)**				**GM**^**#**^	**Median (range)**
5-10	203	1.5	0.5	111.5	111.5^$^	94	2.1	1.1	111.5	111.5^$^	109	0.9	0	0	0
11-15	110	12.7	8.2	24.8	22.0 (9.5-89.0)	52	15.4	11.5	20.0	17.0 (9.5-52.0)	58	10.3	5.2	37.9	33.0 (18.5-89.0)
16-20	57	35.1	10.5	3.8	3.0 (1.0-24.0)	32	43.8	15.6	5.0	4.5 (1.5-24.0)	25	24.0	4.0	1.0	1.0^$^
21-30	113	25.7	5.3	13.5	16.3 (1.0-42.5)	45	33.3	8.9	19.6	16.3 (13.5-42.5)	68	20.6	2.9	6.5	21.5 (1.0-42.0)
31-40	107	25.2	5.6	6.7	9.8 (0.5-189.5)	54	33.3	11.1	6.7	9.8 (0.5-189.5)	53	17.0	0	0	0
41-50	96	25.0	7.3	25.3	23.0 (7.5-230.0)	43	32.6	11.6	29.4	28.5 (7.5-230.0)	53	18.9	3.8	17.3	18.0 (13.0-23.0)
>50	88	19.3	6.8	16.4	18.0 (0.5-203.0)	34	29.4	8.8	8.4	11.5 (0.5-104.5)	54	13.0	5.6	31.9	24.5 (6.5-203.0)
Total	774	17.1	5.3	14.0	15 (0.5-230.0)	35	22.9	8.5	13.2	13.5 (0.5-230.0)	420	12.6	2.6	16.3	23.0 (1.0-203.0)

^#^Geometric mean (GM).

*Infection intensity calculated in the microfilaremic individuals.

^$^Only 1 microfilaremic person in these cells.

**Table 2:** **Univariate analysis of risk factors for *Wuchereria bancrofti *** **antigenemia in Séké Pembé**

	**Total (774)**	**Negative ICT (%)**	**Positive ICT (%)**	***P***	**Males**	**Negative ICT (%)**	**Positive ICT (%)**	***P***	**Females**	**Negative ICT (%)**	**Positive ICT (%)**	***P***
Bed nets												
No	196	155 (79.1)	41 (20.9)	0.123	131	104 (79.7)	27 (20.6)	0.436	65	51 (78.5)	14 (21.5)	0.018
Yes	578	485 (83.9)	93 (16.1)		223	169 (75.8)	54 (24.2)		355	316 (89.0)	39 (11.0)	
Latrines												
No	274	213 (77.7)	61 (22.3)	0.007	128	92 (71.9)	36 (28.1)	0.077	146	121 (82.9)	25 (17.1)	0.042
Yes	500	427 (85.4)	73 (14.6)		226	181 (80.1)	45 (19.9)		274	246 (89.8)	28 (10.2)	
Water source												
River	285	236 (82.8)	49 (17.2)	0.946	131	99 (75.6)	32 (24.4)	0.596	154	137 (89.0)	17 (11.0)	0.458
Public pump	489	404 (82.6)	85 (17.4)		223	174 (78.0)	49 (22.0)		266	230 (86.5)	36 (13.5)	
Hunting and/or fishing*												
No	368	321 (87.2)	47 (12.8)	0.003	134	114 (58.1)	20 (17.9)	0.007	234	207 (88.5)	27 (11.5)	0.549
Yes	370	292 (78.9)	78 (21.1)		200	145 (72.5)	55 (27.5)		170	147 (86.5)	23 (13.5)	
Occasionally sleeping in the bush* (Men only, N = 334)												
No	NA	NA	NA	-	222	190 (85.6)	32 (14.4)	<0.001	NA	NA	NA	-
Yes					112	69 (61.6)	43 (38.4)					
Anti-helminthic drugs within the last year^†^												
No	639	526 (82.3)	113 (17.7)	0.553	292	222 (76.0)	70 (24.0)	0.289	347	304 (87.6)	43 (12.4)	0.760
Yes	135	114 (84.4)	21 (15.6)		62	51 (82.3)	11 (17.7)		73	63 (86.3)	10 (13.7)	
Distance to the field* (km, mean ± standard deviation)	3.6 ± 1.4	3.7 ± 1.4	3.8 ± 1.5	0.535	3.7 ± 1.5	3.7 ± 1.4	3.7 ± 1.5	0.935	3.6 ± 1.4	3.6 ± 1.4	3.8 ± 1.5	0.428

*N = 738 (36 missing data).

^†^1 data missing.

**Table 3:** **Univariate analysis of risk factors for *Wuchereria bancrofti *** **microfilaremia (mf) in Séké Pembé**

	**Total (773**^***#***^**)**	**Negative mf (%)**	**Positive mf (%)**	***P***	**Males**	**Negative mf (%)**	**Positive mf (%)**	***P***	**Females**	**Negative mf (%)**	**Positive mf (%)**	***P***
Bed nets												
No	196	184 (93.9)	12 (6.1)	0.554	131	122 (93.1)	9 (6.9)	0.399	65	63 (95.4)	3 (4.6)	0.273
Yes	577	548 (95.0)	29 (5.0)		222	201 (90.5)	21 (9.5)		355	347 (97.8)	8 (2.2)	
Latrines												
No	273	252 (92.3)	21 (7.7)	0.029	127	112 (88.2)	15 (11.8)	0.094	146	140 (95.9)	6 (4.1)	0.163
Yes	500	480 (96.0)	20 (4.0)		226	211 (93.4)	15 (6.6)		274	269 (98.2)	5 (1.8)	
Water source												
River	285	268 (94.0)	17 (6.0)	0.531	131	117 (89.3)	14 (10.7)	0.257	154	151 (98.1)	3 (1.9)	0.512
Public pump	488	464 (95.1)	24 (4.9)		222	206 (92.8)	16 (7.2)		266	258 (97.0)	8 (3.0)	
Hunting and/or fishing*												
No	368	356 (96.7)	12 (3.3)	0.020	134	128 (95.5)	6 (4.5)	0.034	234	228 (97.4)	6 (2.6)	0.893
Yes	369	343 (92.9)	26 (7.1)		199	177 (88.9)	22 (11.1)		170	166 (97.7)	4 (2.3)	
Occasionally sleeping in the bush* (Men only, N = 334)												
No	NA	NA	NA	-	221	207 (93.7)	14 (6.3)	0.055	NA	NA	NA	-
Yes					112	98 (87.5)	14 (12.5)					
Anti-helminthic drugs within the last year^†^												
No	639	602 (94.2)	37 (5.8)	0.188	292	265 (90.8)	27 (9.3)	0.270	347	337 (97.1)	10 (2.9)	0.462
Yes	134	130 (97.0)	4 (3.0)		61	58 (95.1)	3 (4.9)		73	72 (98.6)	1 (1.4)	
Distance to the field* (km, mean ± standard deviation)	3.6 ± 1.4	3.7 ± 1.5	3.8 ± 1.2	0.570	3.7 ± 1.5	3.7 ± 1.5	3.7 ± 1.5	0.477	3.6 ± 1.4	3.6 ± 1.4	3.9 ± 1.2	0.865

*N = 737 (36 missing data).

^†^1 data missing.

^#^1 positive ICT was not tested for microfilaremia.

### ICT results

**Figure 2:**
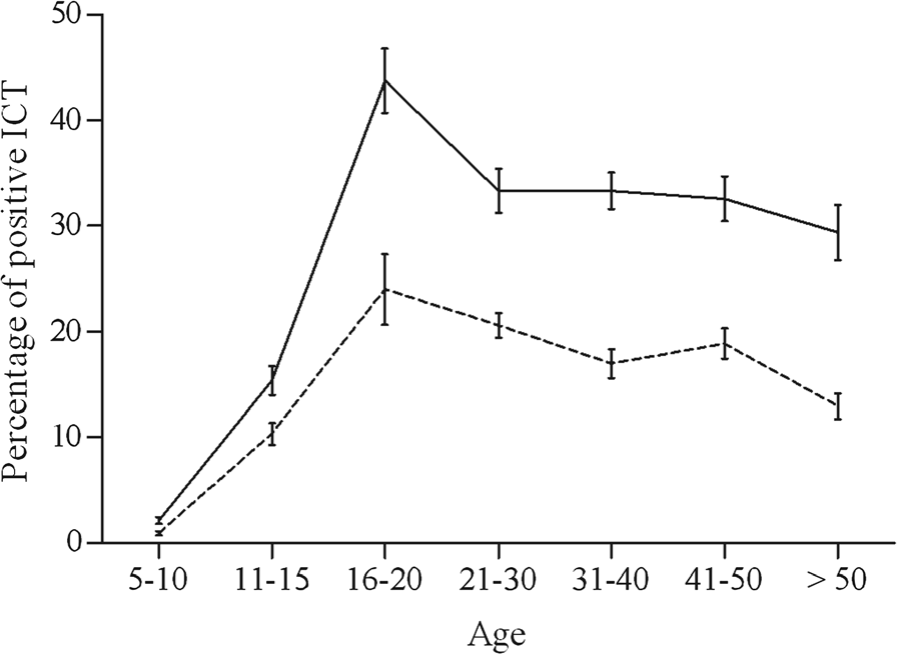
**Age-profiles for *W. bancrofti *antigenemia prevalence.** Males (solid line), females (dashed line). Bars indicate 95% confidence intervals.

The ICT prevalence in the study population was 17.3% (134/774), with a higher value in males than in females (22.9% vs.12.6%, *P* < 0.001). In neighborhoods 1, 2, 3, 4, and 5 there were 14/100 (14.0%), 42/257 (16.3%), 43/221 (19.5%), 23/138 (16.7%), and 12/58 (20.7%) ICT-positive people, respectively (*P* = 0.707). Prevalence rates increased linearly with age for both sexes up to the age class 16-20 years, and then leveled off in males and decreased gradually in females (Figure 2).

### Microfilaremia results

**Figure 3:**
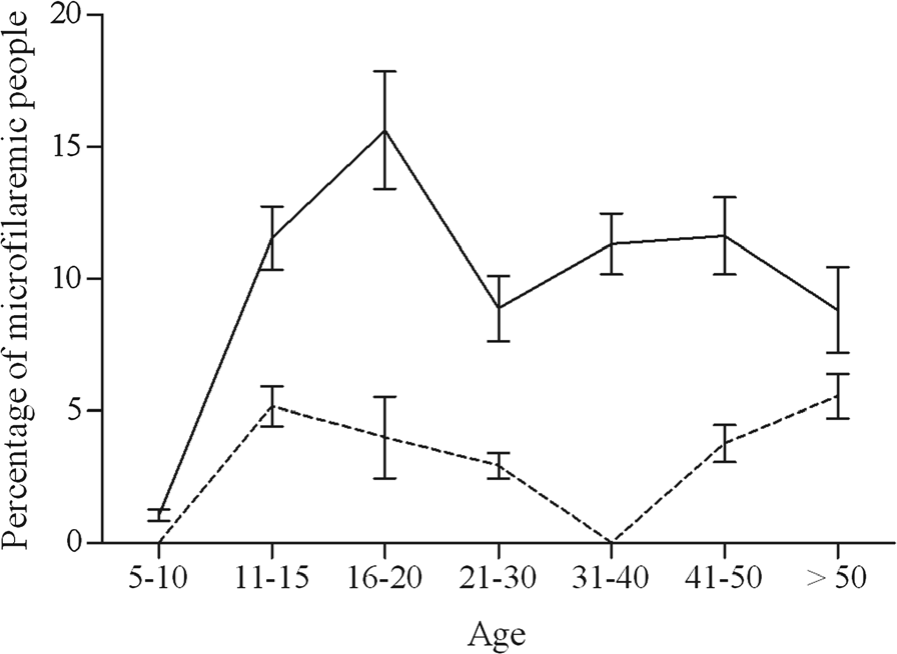
**Age-profiles for *W. bancrofti *microfilarial prevalence.** Males (solid line), females (dashed line). Bars indicate 95% confidence intervals.

*W. bancrofti* mf prevalence was 5.3% (41/774) in the total population and higher in males than in females (8.5% vs. 2.6%, *P* < 0.001). The mf rate in those with positive ICT tests was 30.6%. In neighborhoods 1, 2, 3, 4, and 5 there were 5/100 (5.0%), 11/257 (4.3%), 15/221 (6.8%), 10/138 (7.3%), and 0/58 microfilaremic people, respectively (*P* = 0.174). Microfilaremia rates increased with age for both sexes until age 16-20 years in males and until age 11-15 years in females, and then tended to level off with some fluctuation in older males and females (Figure 3). The arithmetic mean microfilarial density in microfilaremic individuals was 36.8 mf/70 μL (range = 0.5-230.0). It was 35.1 mf/70 μL (standard deviation (sd): 54.7) in males, and 41.3 mf/70 μL (sd: 59.1) in females (*P* = 0.566). The geometric mean microfilarial densities were 14.0, 13.2, and 16.3 mf/70 μL, among microfilaremic subjects of both sexes, males and females, respectively.

### Multivariate models with filarial antigenemia as the dependent variable

In the total population (Table 4), males were twice more likely to be ICT-positive than females (adjusted odds ratio (aOR): 2.0; 95% confidence interval (95% CI) = 1.3-3.0; *P* = 0.002). Subjects aged 11-23 years were much more likely to be infected than those aged 5-10 years (aOR for the 5-10 years: 0.1; 95% CI = 0.0-0.2; *P* < 0.001). The risk for filarial antigenemia did not differ significantly between those aged 11-23 years and the older age groups. Hunting/fishing was associated with an increased risk of infection (aOR: 1.5; 95% CI = 1.0-2.4; *P* = 0.058). In contrast, individuals with a private latrine had a significantly decreased risk for ICT-positivity (aOR: 0.5; 95% CI = 0.4-0.8; *P =* 0.004). Inclusion of a random effect at the household level did not provide any significant improvement in the model (*P =* 0.213). In addition, there was no significant interaction between the covariates after stratifying for gender. For all models, anthelmintic intake within the previous year did not influence the ICT status.

**Table 4:** Multivariate logistic regression analysis of risk factors for filarial antigenemia in the total population

**Variable**	**Adjusted odds-ratio**	**Confidence interval 95%**	***P***
Sex			
Female	1	-	
Male	2.0	1.3-3.0	0.002
Age (years)			
5-10	0.1	0.0-0.2	< 0.001
11-23	1	-	-
24-40	1.6	0.9-2.6	0.081
> 40	1.5	0.9-2.4	0.130
Hunting and/or fishing			
No	1	-	
Yes	1.5	1.0-2.4	0.058
Latrines			
No	1	-	
Yes	0.5	0.4-0.8	0.004

Likelihood-ratio test random effect vs. fixed effect model (*P* = 0.213). N = 738.

Male subjects aged 11-23 years had a higher risk for ICT positivity than those aged 5-10 years, and this risk did not increase further in the older age groups (Table 5). Among the other explanatory variables, the presence of a private latrine had a protective effect (aOR: 0.5; 95% CI = 0.3-0.9; *P =* 0.022). A history of sleeping outdoors was also significantly associated with infection (aOR: 1.9; 95% CI = 1.1-3.5; *P =* 0.028). The ICT status in males was not influenced by the random effect at the household level (*P* = 1.000).

**Table 5:** Multivariate logistic regression analysis of risk factors for filarial antigenemia in the male population

**Variable**	**Adjusted odds-ratio**	**Confidence interval 95%**	***P***
Age (years)			
5-10	0.1	0.0-0.4	0.001
11-23	1	-	-
24-40	1.3	0.6-2.6	0.483
> 40	1.2	0.6- 2.5	0.650
Occasionally sleeping in the bush			
No	1	-	
Yes	1.9	1.1-3.5	0.028
Latrines			
No	1	-	
Yes	0.5	0.3-0.9	0.022
Bed nets			
No	1	-	
Yes	1.3	0.7-2.4	0.410
Intraclass correlation for household			0.0

Likelihood-ratio test random effect vs. fixed effect model (*P* = 1.000). N = 334.

In females, the infection rate peaked at 10-23 years and leveled off after 23 years (Table 6). The use of bed nets had a protective effect (aOR: 0.4; 95% CI = 0.1-0.9; *P =* 0.024). The logistic model with random effect fitted better than the model with fixed effect (Log likelihood = -142.1 for random effect model, and Log likelihood = -143.3 for fixed effect model; *P =* 0.062), and a household effect accounted for 24% of the variation in the ICT rates among females.

**Table 6:** Multivariate logistic regression analysis of risk factors for filarial antigenemia in the female population

**Variable**	**Adjusted odds-ratio**	**Confidence interval 95%**	***P***
Age (years old)			
5-10	0.04	0.0-0.4	0.004
11-23	1	-	-
24-40	1.7	0.7- 4.1	0.269
> 40	1.2	0.5- 3.0	0.654
Bed nets			
No	1	-	
Yes	0.4	0.1-0.9	0.024
Intraclass correlation for household			0.24

Likelihood-ratio test random effect vs. fixed effect model (*P* = 0.062). N = 420.

### Multivariate models with microfilaremia as the dependent variable

Age, sex and latrines were significantly associated with microfilaremia in the total population analysis (Table 7). The risk increased significantly between 5-10 and 11-23 years but did not increase thereafter. Males had a 5-fold increased risk of having mf compared to females (aOR: 5.4; 95% CI = 2.1-13.4; *P* < 0.001), and use of private latrines was associated with a decreased risk of mf (aOR: 0.4; 95% CI = 0.1-0.9; *P =* 0.035). The random effect set at the household level was significant (*P* < 0.001) and explained 48.8% of the variation between individuals for the presence of mf. Gender-stratified multivariate analysis was not performed because very few individuals harbored mf, which provided insufficient statistical power to estimate associations. Lastly, anthelmintic intake within the previous year was not significantly associated with mf.

**Table 7:** **Multivariate logistic regression analysis of risk factors for *W. bancrofti *** **microfilaremia (mf), total-population model**

**Variable**	**Adjusted odds-ratio**	**Confidence interval 95%**	***P***
Sex			
Female	1	-	
Male	5.4	2.1-13.4	<0.001
Age (years old)			
5-10	0.03	0.0-0.3	0.004
11-23	1	-	-
24-40	0.8	0.3- 2.3	0.741
> 40	1.3	0.5- 3.5	0.595
Latrines			
No	1	-	
Yes	0.4	0.1-0.9	0.035
Bed nets			
No	1	-	
Yes	1.2	0.4-3.0	0.764
Intraclass correlation for household			0.488

Likelihood-ratio test random effect vs. fixed effect model (p < 0.001). N = 774.

## Discussion

Data on LF in Central Africa are scarce, and some have questioned whether LF is present in the Republic of Congo [5]. This study has clearly documented that LF is present in that country, which is typical of LF-endemic countries in Central Africa in that it is a post-conflict country with a high burden of loiasis [7]. To our knowledge, this is the first study that has attempted to identify risk factors associated with LF in this region. This type of information may provide insight into factors that affect transmission of *W. bancrofti* in this part of the world.

With ICT and mf prevalence rates of 17.3% and 5.3%, Séké Pembé’s LF rates are the highest that have been confirmed to date in the Republic of Congo. However, these rates are only moderate compared to those reported from untreated populations in many other countries in sub-Saharan Africa [13]. It is interesting that villages a few km from Séké Pembé had much lower LF rates (authors’ unpublished observations). This illustrates the focality of LF in Central Africa. Reasons for this focality remain to be discovered.

The high ratio between ICT and mf rates in this study (3.5) is noteworthy, because this ratio is usually lower in populations that have not received MDA [14]. Widespread informal use of anthelmintic medications could explain this finding. However, our analyses showed that intake of anthelmintic drugs within the last year was not significantly associated with filarial antigenemia or mf status. In addition, most persons who were able to name the drug they had taken mentioned mebendazole, which has very little effect on *W. bancrofti* microfilaremia [15].

The rapid increase in the prevalence rates for filarial antigenemia and microfilaremia during the 20 first years of life may be explained by altered risk behaviors associated with age. The stabilization of infection rates in older age groups is consistent with the meta-analysis of Stolk *et al.*[13]. Males had higher rates of infection than females in Séké Pembé. This pattern is common but not universal in other LF endemic areas [16-26], and it has been reported from many regions [27-34]. Hormonal differences in women have been proposed to explain this difference [35,36].

The use of bed nets was protective for females but not for males in this study. This phenomenon has also been observed in an urban area in Brazil [37]. It is possible that females use bed nets more consistently than males. However, this finding would also be consistent with the hypothesis that males often acquire infections when they are away from home, while women are more often infected in or near their houses. The presence of latrines in the peridomestic environment has been identified as a positive risk factor for *W. bancrofti* infection in areas where *Culex* sp. is the vector [38]. In contrast, use of private outdoor latrines was a negative risk factor for LF in this study area, which presumably has *Anopheles*-mediated transmission. As latrines are close to houses, their use may decrease the risk of infection with *W. bancrofti* by reducing the time when humans are exposed to *Anopheles* mosquitoes outdoors during the vector’s peak feeding times in the evening and night.

A household effect was evident for filarial infections in females (both ICT and mf), but this was not the case for males (ICT). Several elements may contribute to the household effect in females: the habitation type, income level, education level, sanitation, occupation, and the peridomestic environment. Several studies have shown that low socio-economic level [17-20,22-26,39] and low income [27,29,31,34,40,41] are risk factors for LF, probably because they are associated with increased exposure to *Culex* mosquitoes (e.g., in Brazilian slums) [35-37]. However, in one study conducted in India, income level was no longer significantly associated with LF when environmental factors were considered [40]; this supports the hypothesis that the influence of the peridomestic environment predominates in areas with focal or heterogeneous infection [40,42-44]. Since the peridomestic environment was fairly homogenous in Séké Pembé, it plays a marginal role in this village.

The absence of a household effect associated with ICT positivity for males makes the hypothesis of a familial predisposition suggested by Wahyuni *et al.* unlikely [45]. Individual behavior and high rates of mosquito exposure away from their homes appear to be the dominant factors behind high filarial antigenemia rates in males. In contrast, females are probably more likely to be infected near their homes. On the other hand, household was significantly associated with mf status for both sexes.

Sleeping in the bush was an interesting and important risk factor for filarial infection in this study. This risk did not seem related to the activity of hunting or fishing, *per se*. Indeed, males and females who engaged in these activities during the day were not at greater risk for LF. According to the villagers, males who hunt or fish during the day usually come back home before sunset, and this may explain the lack of risk associated with these activities. However, sleeping outside in the bush was always linked to hunting and fishing activities. As the variable “sleeping outside in the bush” could not be entered in the model for both sexes (too few females reported this activity), hunting and fishing tended to be associated with the risk of LF. However, the risk of infection with *W. bancrofti* is probably more closely associated with the time of the activity (i.e. evening and night) and not to the type of activity itself. Although many studies have reported that certain occupations (farmers, plantation workers, fishermen) were associated with an increased risk of LF [19,21,27,31,37,40,43,46-53], the time of day or night when this work takes place can also be very important. When specific activities are associated with intense exposure to mosquito bites, LF can be regarded as an occupational disease, as reported from the Philippines, where males working in abaca plantations had a higher risk of infection than others in the population [41]. More importantly, these occupational or behavioral risks occur outside of the areas where most people live. Individuals who are infected outside of the village can serve as a source of infection for people who live and work inside the village. This is consistent with our hypothesis of peridomestic transmission of filarial infections to females. As suggested in a report on LF in Samoa, we believe that men living in Séké Pembé often acquire LF when they visit a “wild ecologic niche”. Then, they carry the infection back to the village where less intense transmission occurs in the peridomestic setting [46].

A high percentage of males who reported sleeping outside in the bush (almost exclusively for hunting or fishing) on a regular basis (38.4%) were ICT positive compared to 14% ICT positivity in those who never slept outside. The increased risk in the former group probably reflects increased mosquito bites and relatively high rates of infection in others who hunt and fish in the same areas. While natural *W. bancrofti* infection has never been reported in any animal species, patent infections have been established experimentally in three species of langur [54-57] and in *Macaca* monkeys [58,59]. Thus, although unlikely, the possibility of a primate reservoir for *W. bancrofti* in Central Africa cannot be entirely ruled out.

WHO has recently recommended a provisional strategy for eliminating LF from areas where LF is co-endemic with loiasis that includes albendazole MDA with integrated vector management [60]. As LF has been assumed to be absent from the Republic of Congo [5], no study on the vectors of LF has been conducted in the country to date. Very preliminary observations suggest that the vast majority of mosquitoes in Séké Pembé are *Anopheles* sp., mostly *An. funestus* (authors’ unpublished observations). This is consistent with old observations from what is now Bas-Congo province in the Democratic Republic of the Congo [61]. Regarding the Republic of Congo, maps showing the distribution of the various *Anopheles* species have been produced in the 1960s [62] but no update was done since these times. Since local conditions are important for designing IVM interventions [60], additional entomologic studies are needed to better characterize the transmission patterns of *W. bancrofti* in Central Africa.

## Conclusions

This study has provided new information on the epidemiology of lymphatic filariasis in Central Africa, a region that is strategically important for the GPELF. Individual behavioral factors appear to have a major impact on the epidemiology of LF in Séké Pembé; additional studies are needed to understand the dynamics of LF transmission both within the village and in nearby hunting and fishing areas. More broadly, specific behaviors and activities that affect exposure to mosquitoes may also contribute to the highly focal distribution of lymphatic filariasis in Central Africa. Finally, we believe that improved understanding of interactions between human behavior and filariasis transmission could be useful for refining GPELF strategies in ways that may improve the program’s chances for success.

## Abbreviations

GPELF: Global Programme to Eliminate Lymphatic Filariasis; MDA: Mass drug administration; LF: Lymphatic filariasis; IVM: Ivermectin; ALB: Albendazole; NTD: Neglected tropical diseases; WHO: World Health Organization; ICT: Immunochromatographic card test; CFA: Circulating filarial antigens; mf: Microfilariae; DOLF: Death to Onchocerciasis and Lymphatic Filariasis.

## Competing interests

The authors declare that they have no competing interests.

## Authors’ contributions

CBC, FM, SDS, JB, FL, ACM, GJW, and MB participated in the field study. CBC, SDS, PUF, GJW, and MB participated in the redaction of the manuscript. CBC, SDS, ACM, GJW, and MB designed the study. CBC, SDS, GJW, and MB analyzed data. All authors read and approved the final version of the manuscript.
